# Designer Spin Models in Tunable Two-Dimensional Nanographene Lattices

**DOI:** 10.1021/acs.nanolett.3c04915

**Published:** 2024-03-01

**Authors:** João Henriques, Mar Ferri-Cortés, Joaquín Fernández-Rossier

**Affiliations:** † International Iberian Nanotechnology Laboratory (INL), Av. Mestre José Veiga, 4715-330 Braga, Portugal; ‡ Universidade de Santiago de Compostela, 15782 Santiago de Compostela, Spain; ¶ Departamento de Física Aplicada, Universidad de Alicante, 03690 San Vicente del Raspeig, Spain

**Keywords:** nanographenes, star lattice, valence bond crystal, triplons

## Abstract

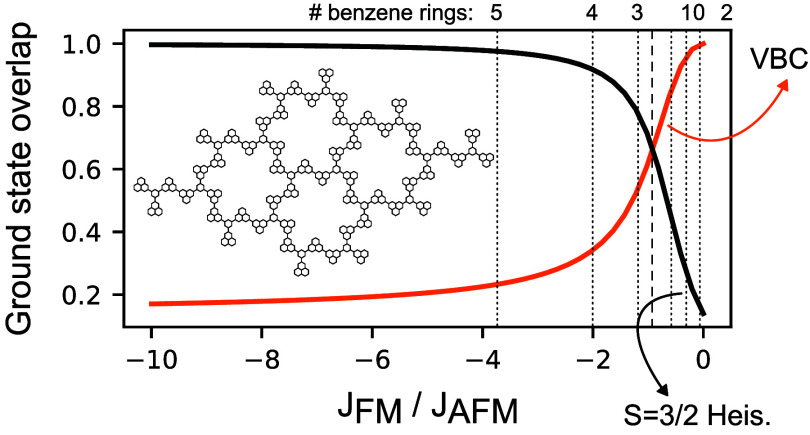

Motivated by recent experimental breakthroughs, we propose a strategy for designing two-dimensional spin–lattices with competing interactions that lead to nontrivial emergent quantum states. We consider *S* = ^1^/_2_ nanographenes with *C*_3_ symmetry as building blocks, and we leverage the potential to control both the sign and the strength of exchange with first neighbors to build a family of spin models. Specifically, we consider the case of a Heisenberg model in a triangle-decorated honeycomb lattice with competing ferromagnetic and antiferromagnetic interactions whose ratio can be varied in a wide range. On the basis of the exact diagonalization of both Fermionic and spin models, we predict a quantum phase transition between a valence bond crystal of spin singlets with triplon excitations living in a Kagomé lattice and a Néel phase of effective *S* = ^3^/_2_ in the limit of dominant ferromagnetic interactions.

Artificial lattices, created through precision engineering at the nanoscale, can serve as powerful tools for investigating emergent quantum phenomena across various physical platforms, such as ultracold atoms in optical lattices,^1^ trapped ions,^2^ Rydberg atoms,^3^ superconducting circuits,^4^ semiconductor quantum dots,^5^ and magnetic adatoms.^6^ These lattices mimic the periodic structures found in condensed matter systems and can help us to emulate and understand exotic quantum states, predicted by theoretical models,^7,8^ that may be challenging to observe directly in natural materials and can play a crucial role in the development of quantum coherent nanotechnology.^9^ Here, we propose to leverage the gigantic know-how of organic chemistry using open-shell nanographenes as building blocks to create artificial quantum matter.^10^ Open-shell nanographenes are planar carbon molecules with a finite spin *S* in the ground state. The formation of supramolecular structures, such as dimers,^11,12^ trimers,^13^ rings,^14^ chains,^15^ and two-dimensional arrays,^16,17^ that preserve the open-shell nature of the building blocks^18^ has recently been demonstrated. This bottom-up approach permits one to design^18−20^ two-dimensional carbon crystals with very narrow bands, linear combinations of carbon p_*z*_ orbitals, and strong electron correlations, complementing the top-down approach of magic angle twisted bilayer graphene.^21,22^

In the case of one-dimensional nanographene chains, quantum fluctuations destroy long-range order, and outstanding quantum magnetism phenomena, such as the valence bond solid (VBS)^23^ with fractional edge states in *S* = 1 chains^15^ and the topological dimerized phase of the alternate bond *S* = ^1^/_2_ chain,^24^ have been reported. In two dimensions, spin–lattices can display either broken symmetry phases with magnetic order and gapless spin excitations as well as gapped quantum disordered ground states, some of which can have emergent properties, and have potential for applications in quantum information.^7,8^

The properties of both ground state and spin excitations depend crucially on the lattice as well as the sign, symmetry, and strength of the interactions therein. For instance, experiments show that *S* = ^3^/_2_ triangulenes naturally form honeycomb lattices,^17^ and theory shows^18^ that these are described with nonlinear Heisenberg spin models that belong to the same class of the AKLT Hamiltonian^23^ with a VBS ground state. However, calculations^18^ show that the nonlinear exchange is way too small in this system when compared with that of the AKLT model, which very likely will drive the system toward a Néel ground state^25^ with gapless magnon excitations.

Here we pose the challenge of engineering a planar nanographene lattice that, depending on structural design parameters, can display either a broken symmetry ground state with gapless spin excitations or a quantum disordered ground state with a gapped spectrum. For that matter, we propose a system that realizes the so-called star lattice,^26^ a triangle-decorated honeycomb graph, with *S* = ^1^/_2_ spins (See Figure 1). We choose as our *S* = ^1^/_2_ units phenalenyl molecules,^27^ known to form open-shell dimers^12^ and trimers.^13^ In each site of the honeycomb lattice, we place three phenalenyls, coupled via a benzene linker that, as we show, induces a weak ferromagnetic exchange. Each phenalenyl in a trimer is coupled with one additional molecule from a different site of the honeycomb lattice, in a configuration known to give antiferromagnetic exchange.^12,28^

**Figure 1:**
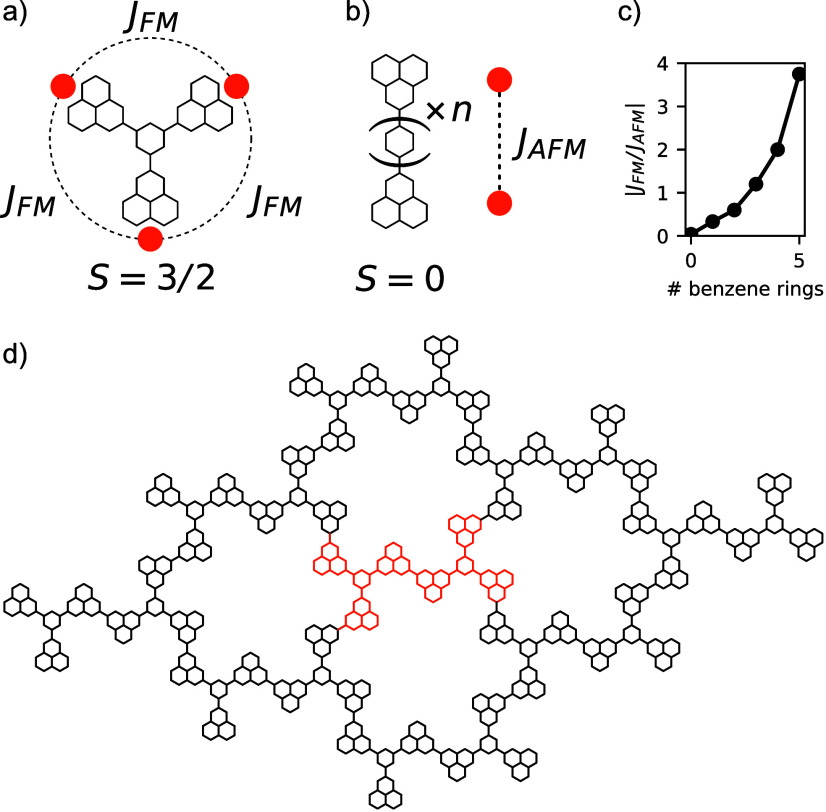
Schematic representation of (a) a phenalenyl trimer and (b) a phenalenyl dimer with different numbers of benzene rings, *n*, between the two molecules. The effective *S* = ^1^/_2_ spin models and the predicted spin of the many-body ground state according to Lieb’s theorem are also shown. (c) Ratio of the trimer ferromagnetic and dimer antiferromagnetic exchange for different numbers of benzene rings in the dimer (results obtained from a CAS calculation with the four least energetic orbitals in the Hilbert space). (d) Depiction of a fraction of a two-dimensional crystal obtained by combining phenalenyl trimers. The unit cell is colored orange.

We start our discussion with the introduction of the Hubbard model, which we shall use to describe the phenalenyl dimers and trimers considered in this work. The use of the Hubbard model to model planar open-shell nanographenes is known to yield good agreement with both quantum chemistry^29,30^ and experimental results.^11,15^

Explicitly, the model reads^31^
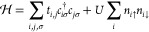
1where the indices *i* and *j* run over carbon atoms (which are the first and third neighbors, only), *t*_*i*,*j*_ stands for the hopping between sites *i* and *j*, and *U* is the on-site Hubbard repulsion. In the rest of the paper, we shall consider the first neighbor hopping to be *t* = −2.7 eV and the third neighbor hopping to be *t*_3_ = *t*/10 and we set the Hubbard repulsion to *U* = |*t*|, which has been shown to agree well with *ab initio* results from previous works.^18,32^ The operators *c*_*iσ*_^†^ (*c*_*iσ*_) represent the creation (annihilation) of an electron at site *i* with spin projection along a quantization axis σ = *↑*, *↓*, and *n*_*iσ*_ = *c*_*iσ*_^†^*c*_*iσ*_ is the corresponding number operator.

For a phenalenyl molecule, due to its sublattice imbalance of 1 (one sublattice contains one additional atom when compared with the other), a single mode at zero energy is expected in its single-particle spectrum.^33^ Accounting for the Hubbard repulsion, Lieb’s theorem^34^ predicts a many-body ground state with an *S* = ^1^/_2_ spin, in agreement with the Ovchinnikov rule.^35^ This has been verified experimentally^36^ and theoretically^37,38^ in previous works.

When phenalenyl molecules are combined to form supramolecular structures, such as dimers or trimers (see Figure 1a,b), Lieb’s theorem^34^ predicts that the many-body ground state will have a spin that is larger or smaller than ^1^/_2_ depending on how the molecules are connected. In particular, if three phenalenyl molecules are linked together via a central benzene ring, preserving *C*_3_ symmetry, Lieb’s theorem states that the three *S* = ^1^/_2_ spins couple ferromagnetically, leading to a many-body ground state with an *S* = ^3^/_2_ spin; this is a consequence of the sublattice imbalance increasing to 3 and can be microscopically explained via Hund’s ferromagnetic exchange between the zero modes hosted by the three phenalenyl building blocks.^28,30^

To estimate the magnitude of the effective ferromagnetic interaction that leads to an *S* = ^3^/_2_ ground state, we solve the Hubbard model for the trimer of Figure 1a using the configuration interaction (CI) method in the complete active space (CAS) approximation, where the Hilbert space is restricted to the three single particle states at zero energy^30^ (see the Supporting Information). Doing so, we find that, in agreement with Lieb’s theorem, the molecule presents a quartet ground state corresponding to an *S* = ^3^/_2_ spin; the first excited state is also a quartet, made of two degenerate doublets. On the basis of the low-energy spectrum of the CAS calculation, we postulate a spin model to describe this system, consisting of three *S* = ^1^/_2_ spins coupled ferromagnetically all-to-all, with an exchange coupling given approximately by *J*_FM_ ≈ −3 meV.

As opposed to the trimer, the phenalenyl dimer has no sublattice imbalance, and as a consequence, its many-body ground state is a singlet. Microscopically, this implies that intermolecular exchange is antiferromagnetic^12,28^ and favors low-spin ground states. Performing a CAS calculation for the dimer (where only the zero modes of the two phenalenyls are accounted for in the Hilbert space), one indeed finds a singlet ground state split by ∼63 meV from a triplet excited state (see the Supporting Information), in qualitative agreement with the results of recent experiments;^12^ at higher energies, well separated from these low-energy states, other excitations appear. The results of the multiconfigurational calculation lead us to describe this molecule as having two antiferromagnetically coupled *S* = ^1^/_2_ spins with an antiferromagnetic exchange of *J*_AFM_ ≈ 63 meV. Importantly, by introducing benzene rings between the molecules, our calculations show that the magnitude of the antiferromagnetic intermolecular exchange is reduced dramatically (see Figure 1c). This reduction has also been reported in the case of *S* = 1 triangulene dimers.^11^

On the basis of the analysis we have just performed, one sees that if two phenalenyl trimers are combined in the way depicted in Figure 1d, then it is possible to build a two-dimensional nanographene crystal with competing ferromagnetic and antiferromagnetic interactions in the unit cell. Because we have argued that the building blocks of such a crystal are described well by spin models, it is only natural to extend the same reasoning to the crystal. Hence, we propose the following *S* = ^1^/_2_ Heisenberg Hamiltonian in the star lattice to describe the structure depicted in Figure 1d:
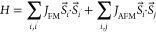
2where *i* and *i*′ are first neighbors belonging to the same trimer while *i* and *j* are first neighbors that belong to adjacent trimers. The values of *J*_FM_ and *J*_AFM_ are obtained from our Hubbard–CAS calculations for the trimer and dimer, respectively. In Figure 2a, we depict the spin model for the crystal, where the geometry of a star lattice is evident. A variation of this model, in which Kitaev exchange is also included, was considered in ref (39).

**Figure 2:**
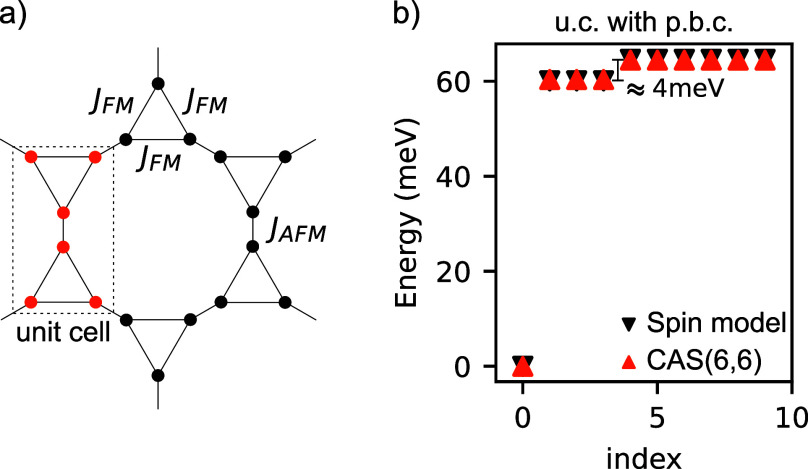
(a) Schematic representation of the spin model for the crystal, forming a star lattice. The dashed rectangle highlights the unit cell of the crystal. (b) Comparison between the results obtained from CAS(6,6) and exact diagonalization of the spin model for the unit cell (u.c.) of the crystal with periodic boundary conditions (p.b.c.).

To validate the proposed spin model, we compared the results obtained from exact diagonalization for the unit cell of the crystal with periodic boundary conditions to those obtained with CAS for the same structure for the case of zero benzene spacers in the dimer. Excellent agreement between the two approaches is seen in Figure 2b, thus validating the proposed spin model. In the CAS calculation, only the six single particle states closer to zero energy were considered, as these are expected to make the largest contribution to the low-energy part of the many-body spectrum. Even though we account for only exchange interactions between first neighbors, we could, in principle, consider interactions between second neighbors and beyond. For the system presented here, the great agreement between the spin and Fermionic models (see Figure 2b) shows that interactions beyond first neighbors are very weak. However, in other star lattice systems with second neighbor exchange that promotes frustration, for instance, new phases could appear.

The proposed star lattice model has two competing interactions, *J*_FM_ and *J*_AFM_, whose ratio can be controlled, in the proposed system, by changing the number of benzene spacers, *n*, in the phenalenyl dimers. We now discuss two limiting cases of the model: *J*_FM_ = 0 and *J*_AFM_ = 0. In the former, the star lattice becomes a Kagomé lattice of independent pairs of antiferromagnetically coupled phenalenyl molecules. The ground state wave function in this case is a valence bond crystal (VBC), with an excited manifold of 3*N* degenerate *S* = 1 excitations with energy *J*_AFM_, where *N* is the number of unit cells in the crystal. When *J*_FM_ is turned on but remains much smaller than *J*_AFM_, the ground state remains close to a VBC and the *S* = 1 excited states acquire a dispersion, forming triplon bands (more on this below); this explains the energy splitting seen in the excited manifold in Figure 2b. Solving the model analytically, one finds the splitting of the first excited manifold to be given by 3*J*_FM_/2, in good agreement with the numerical result. In the opposite limit, i.e., *J*_AFM_ = 0, we find 2*N* independent trimers with *S* = ^3^/_2_ spin. When *J*_AFM_ is turned on, we are left with a honeycomb lattice of *S* = ^3^/_2_ spins with antiferromagnetic interactions. This broken symmetry limit has been discussed by Owerre,^40^ who found topological magnons.

The star lattice Hamiltonian has been studied before in the case in which both interactions are antiferromagnetic,^26,41−43^ where spin frustration in the triangles can promote spin-liquid behavior, consistent with recent experimental observations.^44^ The case with intratriangle antiferromagnetic and intertriangle ferromagnetic exchange couplings has also been studied.^45^ In our case, however, the interactions within each triangle are ferromagnetic instead and antiferromagnetic exchange links different triangles. To infer the properties of this system, we now exactly diagonalize the spin model for a ring made of six trimers, containing a total of 18 spins, with periodic boundary conditions (see Figure 2a). We focus on two aspects: the nature of the ground state wave function and the low-energy excitation spectrum.

To gain insight into the nature of the ground state of this system, we compute the overlap between its wave function and another two particularly relevant states: (i) the wave function of the VBC and (ii) the ground state of the *S* = ^3^/_2_ antiferromagnetic Heisenberg model. For the first case, we simply build the VBC wave function by solving the spin model with *J*_FM_ = 0 and then compute the overlap with the solution for several values of *J*_FM_/*J*_AFM_. With regard to the second case, we solve the *S* = ^3^/_2_ Heisenberg model that we then project into a spin-1/2 basis, thus allowing the computation of the overlap with the wave function of our system; the results are displayed in Figure 3a. There, we see that as expected, the overlap between the exact ground state wave function and that from the VBC starts at 1 for *J*_FM_ = 0 and quickly decreases as the ratio of ferro- and antiferromagnetic interactions increases. On the contrary, the overlap with the *S* = ^3^/_2_ Heisenberg model wave function starts at ∼0.14 and increases with |*J*_FM_/*J*_AFM_|. In the limit |*J*_FM_| ≫ *J*_AFM_, the overlap approaches 1, as expected, given the physical picture presented before. Crucially, by controlling the molecular linking groups, one seems to be able to induce a quantum phase transition in the system, as its ground state changes from a quantum disordered one in the VBC limit to a broken symmetry one in the Heisenberg limit. The broken symmetry order parameter for such a phase, computed with the mean-field Hubbard model, is shown in the Supporting Information for two different values of *J*_FM_/*J*_AFM_.

**Figure 3:**
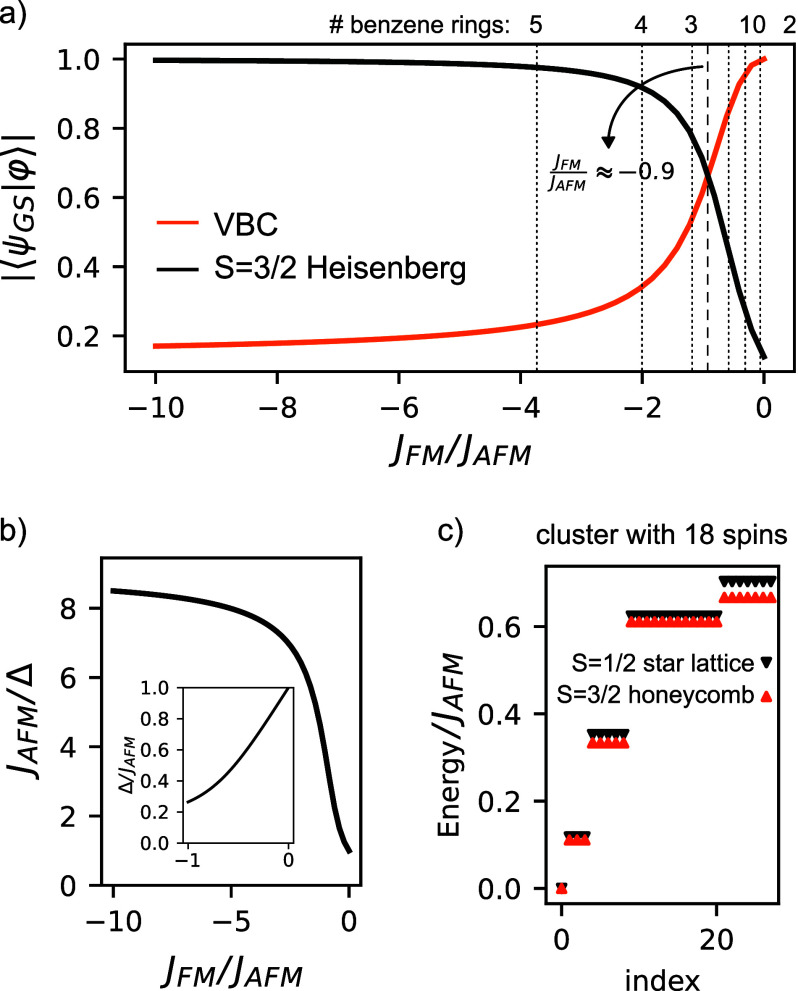
(a) Overlap between the wave function of the ground state of a ring of six trimers, with a total of 18 spins, with p.b.c., |ψ_GS_⟩, and the wave functions of the valence bond crystal (VBC) and the *S* = ^3^/_2_ Heisenberg model ground state for the same geometry. The dashed vertical line marks the point at which the two overlaps are identical, and the dotted lines refer to the values of *J*_FM_/*J*_AFM_ for different numbers of benzene spacers in the phenalenyl dimers (cf. Figure 1c). (b) Energy of the first excitation, Δ, as a function of *J*_FM_/*J*_AFM_. (c) Exact diagonalization of a spin model of the six-trimer ring with *J*_FM_ = −10*J*_AFM_ for the *S* = ^1^/_2_ star lattice and  for the *S* = ^3^/_2_ honeycomb geometry.

In panels b and c of Figure 3, we focus on the excitation spectrum obtained via exact diagonalization of the spin model for the 18-spin cluster we have just considered. For all considered values of *J*_FM_/*J*_AFM_, we find a singlet ground state, followed by a triplet excitation. In panel b, we plot the energy of the first excited manifold, Δ, as a function of the ratio of ferro- and antiferromagnetic interactions. In the limit |*J*_FM_|/*J*_AFM_ ≪ 1, where the system is close to a VBC with a quantum disordered ground state, the gap of the system is essentially *J*_AFM_ and the first set of excited states corresponds to a set of almost degenerate triplets, slightly split due to the finite *J*_FM_. As |*J*_FM_|/*J*_AFM_ increases and the system undergoes a transition from a VBC to an *S* = ^3^/_2_ Heisenberg model, the gap of the first excitation decreases sharply and saturates at . The finite value of this gap is due to the finite size of our simulation lattice. The relation between  and *J*_AFM_ is identical to that expected from a classical model (see the Supporting Information). In Figure 3c, we compare the low-energy spectrum of the *S* = ^1^/_2_ star lattice, using *J*_FM_ = −10*J*_AFM_, with the energy spectrum of an *S* = ^3^/_2_ honeycomb Heisenberg model with exchange . There one finds that the two data sets are in good agreement; we have verified that as we approach the limit |*J*_FM_|/*J*_AFM_ → *∞* an exact agreement appears (see the Supporting Information).

We now focus on the excitations in the small |*J*_FM_|/*J*_AFM_ limit. These excitations are triplons, which correspond to *S* = 1 excitations that propagate in the Kagomé lattice defined by the bonds of the honeycomb lattice of antiferromangetically coupled phenalenyls. In this limit, we can employ the mean-field self-consistent bond operator formalism introduced by Sachdev and Bhatt.^46−48^ Within this framework, we assign a singlet and three triplet creation operators to each antiferromagnetically coupled dimer of the unit cell. Expressing the Hamiltonian of the spin model in terms of these bosonic bond operators and diagonalizing it via a paraunitary transformation,^49^ we find the triplon dispersion depicted in Figure 4 (see the Supporting Information). These bands follow the usual dispersion of Kagomé systems, with two dispersive bands and a single flat one. This set of three bands is centered approximately at the energy of the antiferromagnetic exchange, and the bandwidth is controlled by ferromagnetic coupling. Reducing the strength of the antiferromagnetic coupling, while still keeping it larger than the ferromagnetic interaction, simply leads to a downward shift of the band structure; further decreasing *J*_AFM_ would cause an instability in the triplon spectrum, indicating the transition from a quantum disordered ground state to a broken symmetry one.

**Figure 4:**
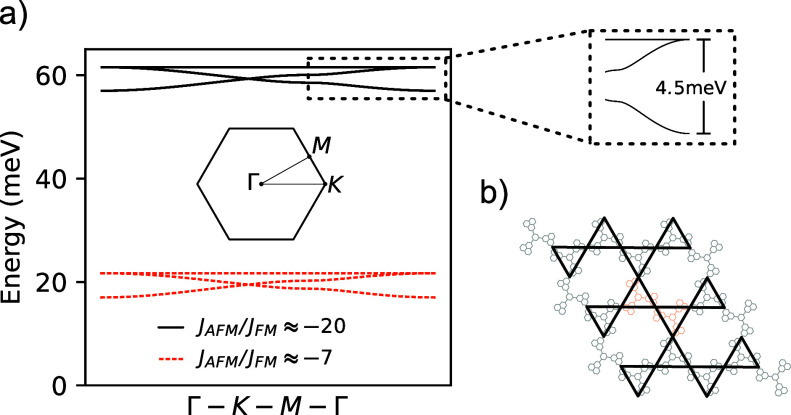
(a) Triplon dispersion obtained with the mean-field self-consistent bond operator formalism. Solid black lines show the bands for the *J*_AFM_ = 63 meV case, and dashed orange lines refer to the *J*_AFM_ = 20 meV case; in both cases, *J*_FM_ = −3 meV. (b) Schematic representation of the effective Kagomé lattice formed by the phenalenyl pairs that link different trimers.

In summary, we propose a strategy for creating artificial two-dimensional spin–lattices with tunable competing interactions using nanographenes. Specifically, we have shown that phenalenyls can be used as building blocks to realize the *S* = ^1^/_2_ star lattice model with competing ferromagnetic (*J*_FM_) and antiferromagnetic (*J*_AFM_) couplings. We have shown that the figure of merit of this model, the *J*_AFM_/*J*_FM_ ratio, can be tuned in a range that permits one to cross the transition between two very different phases, an *S* = ^1^/_2_ valence bond crystal state with gapped triplons and an *S* = ^3^/_2_ Néel ordered phase. Experimentally, finite size nanographene lattices are to be expected, and the VBC phase should have in gap edge states,^17^ analogous to those of the Haldane chains. Other materials^44,50^ in which the star lattice can be realized have previously been reported, but these lack the tunability offered by nanographenes. Our results illustrate the potential of nanographene-based artificial lattices to apply the gigantic power of synthetic organic chemistry to engineer nontrivial quantum spin states.

## Supporting Information Available

Additional details about the CAS calculation for the dimer and trimer building blocks and for the unit cell of the crystal with periodic boundary conditions, discussion of the Néel order based on mean-field Hubbard calculations and the triplon spectrum calculation, and classical estimation of exchange coupling and comparison between spin models (PDF)
