# Supplementary material for: Designer Spin Models in Tunable Two-Dimensional Nanographene Lattices
Source: Nano Lett. 2024 Mar 1;24(11):3355–60. doi: 10.1021/acs.nanolett.3c04915 (PMC10958603; doi:10.1021/acs.nanolett.3c04915)
Supplement: Supplementary file 1 — nl3c04915_si_001.pdf [file nl3c04915_si_001.pdf]

Supporting Information: Designer spin models in tunable two-dimensional nanographene lattices

João Henriques,[†,‡] Mar Ferri-Cortés,[¶] and Joaquín Fernández-Rossier[*,†,§]

† *International Iberian Nanotechnology Laboratory (INL), Av. Mestre José Veiga, 4715-330 Braga, Portugal*

‡ *Universidade de Santiago de Compostela, 15782 Santiago de Compostela, Spain*

¶*Departamento de Física Aplicada, Universidad de Alicante, 03690 San Vicente del Raspeig, Spain*

§

On permanent leave from Departamento de Física Aplicada, Universidad de Alicante, 03690 San Vicente del Raspeig, Spain

E-mail: joaquin.fernandez-rossier@inl.int

CAS calculation for dimers and trimer

In this section we describe how to solve the Hubbard model using the Complete Active Space (CAS) approximation, and give results for phenalenyl dimers and trimers. The exchange couplings used in the spin models of the main text are obtained from the CAS calculations we show below.

First, we write the Hubbard model as defined in the main text

$$\mathcal{H} = \sum_{i,j,\sigma} t_{i,j} c^\dagger_{i\sigma} c_{j\sigma} + U \sum_i n_{i\uparrow} n_{i\downarrow}, \tag{1}$$

where the indices i, j run over carbon atoms (which are first and third neighbors, only), $t_{i,j}$ stands for the hopping between sites i and j, and U is the on-site Hubbard repulsion. In the following we shall consider the first neighbor hopping to be $t = -2.7$ eV, the third neighbor hopping to be $t_3 = t/10$ and we set the Hubbard repulsion to $U = |t|$, which has been shown to agree well with *ab initio* results in previous works.[1] The operators $c^{\dagger}_{i\sigma}$ ($c_{i\sigma}$) represent the creation (annihilation) of an electron in site i with spin projection along a quantization axis $\sigma = \uparrow, \downarrow$, and $n_{i\sigma} = c^{\dagger}_{i\sigma} c_{i\sigma}$ is the corresponding number operator. While the first term in the Hamiltonian describes hopping between different sites, the second deals with the intra-atomic Coulomb repulsion cost associated to having a given site doubly occupied.

Solving the Hubbard model exactly is only possible for small structures due to the exponential increase of the Hilbert space with the number of sites. To circumvent this problem, the configuration interaction approach combined with the CAS approximation is often employed.[2] In this framework, the single particle problem ($U = 0$) is solved first. Then, the full Hamiltonian (with $U \neq 0$) is expressed in terms of the single particle eigenstates (termed molecular orbitals). Afterwards, N_e electrons are distributed over a subset of N_O orbital close to zero energy, and all possible electron configurations are considered (which we term CAS(N_e, N_O)). The orbitals below the active space are fully occupied with the remaining electrons, while the ones above the active space are empty; both sets of orbitals have their electron occupancy frozen. For nanographenes, where we consider only a singly occupied p_z orbital for each Carbon site, the number of electrons is equal to the number of orbitals. As a result, at charge neutrality, one has $N_e = N_O$. In the following we shall compute the single particle spectrum and solve the Hubbard model within the CAS approximation for different structures obtained by combining phenalenyl molecules.

Starting with the phenalenyl trimer, where three units are combined via a central benzene ring, we obtain the single particle spectrum depicted in Fig. 1a. As expected, due to the sublattice imbalance of this molecule, three states at zero energy are visible, one for each phenalenyl. Solving the Hubbard Hamiltonian with $U = |t|$ in CAS(3,3), where only the three

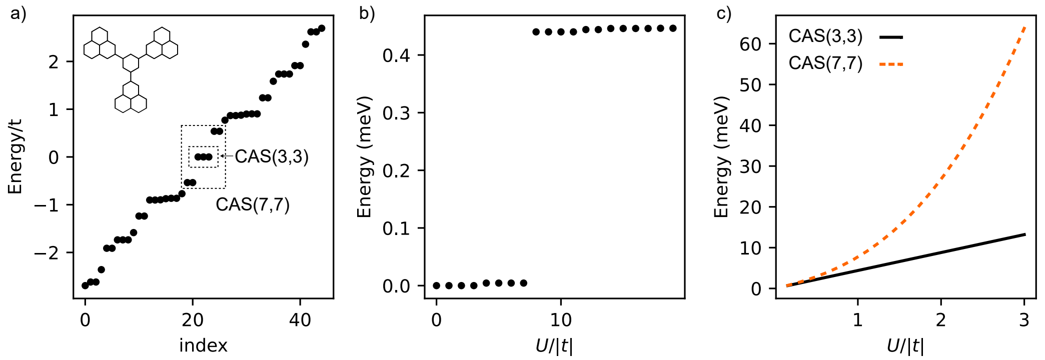

Figure 1: (a) Single particle spectrum for triangulene trimer; (b) CAS(3,3) solution of the Hubbard model with $U = |t|$; (c) Energy of the first excited state as a function of U in two active spaces. In all cases $t = -2.7$eV and $t_3 = t/10$ were considered.

zero modes are accounted for, one finds a quartet ground state (see Fig. 1b), in agreement with Lieb's theorem. The first excited state, which is also a quartet, appears approximately 5 meV above the ground state. Modelling the system with a Heisenberg Hamiltonian where three spin-1/2 are coupled all-to-all via a ferromagnetic exchange $J_{\rm FM}$, we find the energy of the first excitation to be $3J_{\rm FM}/2$. Solving for $J_{\rm FM}$ we find the value introduced in the main text, $J_{\rm FM} \approx -3$ meV. To study the influence of the choice of active space we solve the Hubbard Hamiltonian with CAS(3,3) and CAS(7,7) for several values of U; the results are summarized in Fig. 1c. We see that by including orbitals beyond the zero modes we activate the so-called Coulomb driven super-exchange mechanism,[3] which increases the energy of the first excited state (and hence the ferromagnetic exchange) when compared with the CAS(3,3) calculation. In the main text we ignore this correction as it does not change the qualitative features of the system we focus on.

Let us now consider the case of the dimer, where two phenalenyl molecules are linked vertex to vertex. In Fig. 2a we depict the single particle spectrum and find no modes at zero energy. The zero modes of the two phenalenyl hybridize via third neighbor hopping, leading to the formation of bonding and antibonding orbitals with finite energy in the dimer. This result was to be expected, as a consequence of the lack of sublattice imbalance in the dimer. In Fig. 2b we show the CAS(2,2) result with $U = |t|$. In agreement with

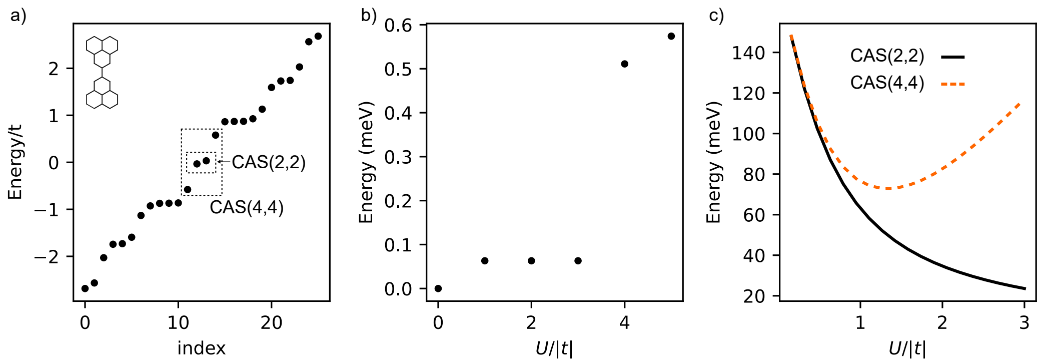

Figure 2: (a) Single particle spectrum for triangulene dimer; (b) CAS(2,2) solution of the Hubbard model with $U = |t|$; (c) Energy of the first excited state as a function of U in two active spaces. In all cases $t = -2.7$eV and $t_3 = t/10$ were considered.

Lieb's theorem the ground state is a singlet split by approximately 60meV from the first excited state, a triplet; at higher energies, well separated from these low energy states, other manifold appear. Modelling the low energy part of the spectrum with a spin model of two antiferromagnetically coupled spin-1/2, we find $J_{\mathrm{AFM}} = 63$ meV. To check the impact of the active space in the calculation we once again solve the Hubbard Hamiltonian in two distinct active spaces for several values of U as depicted in Fig. 2c. We find, once more, that including additional orbitals increases the exchange strength due to the Coulomb driven super-exchange mechanism.[3] Crucially, for $U = |t|$ the singlet-triplet splitting is already well described by the minimal CAS(2,2). Also, comparing the results of the dimer and the trimer, we see that regardless of the chosen active space, one has $J_{\mathrm{AFM}} \gg J_{\mathrm{FM}}$, thus guaranteeing that the qualitative features of the model we studied in the main text should be preserved regardless of the choice of active space.

At last, let us look at the case where two phenalenyl molecules are coupled via a central benzene ring. This situation is similar to the one we have just considered, with the difference that the additional benzene rings weakens intermolecular hybridization, which in turn will be responsible for a smaller antiferromagnetic exchange. From Fig. 3 we see that the zero mode splitting is smaller than in the case without the benzene ring, as expected. Solving the Hubbard model for $U = |t|$ (see Fig. 3b) one finds that the singlet and triplet are now

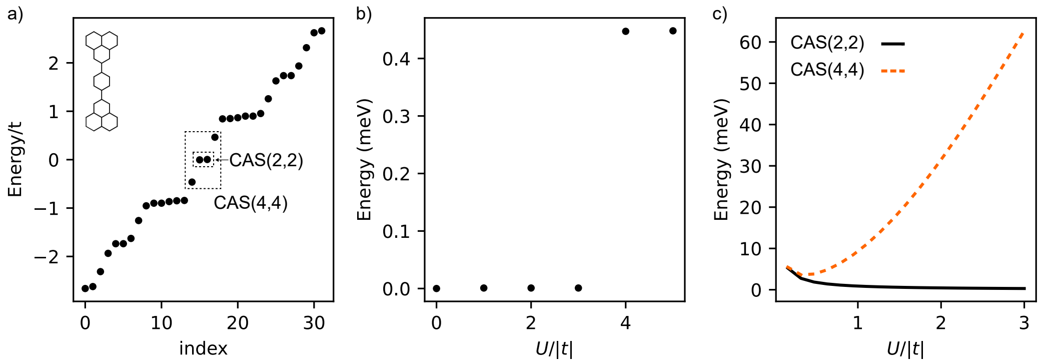

Figure 3: (a) Single particle spectrum for triangulene dimer with a benzene ring between the two phenalenyl; (b) CAS(2,2) solution of the Hubbard model with $U = |t|$; (c) Energy of the first excited state as a function of U in two active spaces. In all cases $t = -2.7$eV and $t_3 = t/10$ were considered.

almost degenerate, reflecting the reduction of the antiferromagnetic exchange. Comparing the singlet-triplet splitting in two active spaces (see Fig. 3c) we see that the Coulomb driven contribution plays a bigger role in this system for $U \sim |t|$ than it did in the case without the benzene ring. Nevertheless, the exchange is still significant smaller than in the previous case, showing that the antiferromagnetic coupling can be easily reduced by progressively decoupling the two phenalenyl molecules with the introduction of benzene rings between them.

Crystal

In this section we show non interacting bands of the crystal discussed in the main text. Using the tight-binding Hamiltonian introduced in the main text (Hubbard model with $U = 0$), we obtain the non-interacting bands depicted in Fig. 4a. At low energy we find 2 pairs of particle-hole symmetric bands, where each pair is made of 3 bands showing a Kagomé dispersion. In the absence of intra-trimer hybridization, the crystal would simply show two sets of particle-hole symmetric flat bands, each set thrice degenerate, close to zero energy, due to the formation of bonding and anti-bonding states between the zero modes of the phenalenyl molecules which are linked vertex to vertex; these states form a virtual Kagomé

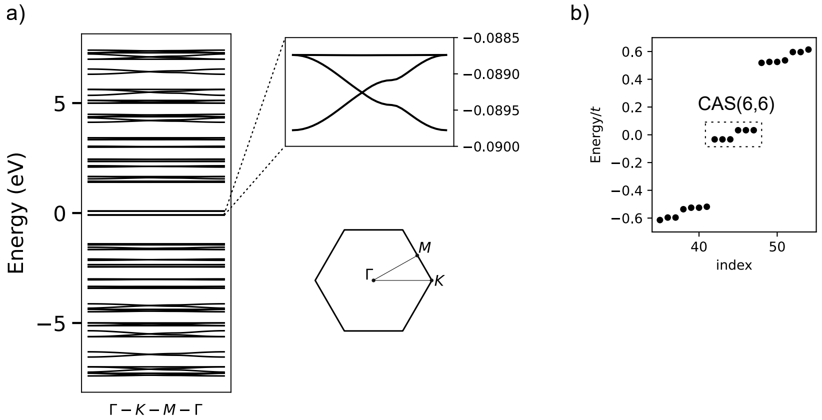

Figure 4: (a) Bands obtained with the tight binding model for the crystal. The inset shows in more detail the three valence bands closest to zero energy; particle-hole symmetric bands appear at positive energy. Also shown is the first Brillouin zone with the relevant high symmetry points. (b) Energies at the Γ point, which correspond to the single particle energies for the unit cell with periodic boundary conditions. The 6 states closest to zero energies form the Hilbert space used in the CAS calculation of the main text, where 6 electrons are distributed over the 6 highlighted states.

lattice. Once the intra-trimer hybridization is turned on, hoppings between the bonding (or anti-bonding) states appear, and the flat bands acquire the dispersion shown in Fig. 4a. The narrowness of these bands can be ascribed to the small strength of the intra-trimer interaction. In Fig. 4b we show the energies at the Γ point, which correspond to the single particle energies for the unit cell with periodic boundary conditions. The 6 states closest to zero energy form the restricted Hilbert space which enters in the CAS calculation shown in the main text.

Néel order from mean-field Hubbard

To gain insight about the broken symmetry solutions of our system we use a mean-field approach (which always gives broken symmetry solutions) to solve the Hubbard Hamiltonian for the unit-cell of the crystal with periodic boundary conditions, for two different cases: i) two benzene spacers, and ii) four benzene spacers between the phenalenyl dimers. These two situtation correspond to two different ratios of J_{FM} and J_{AFM} in the corresponding

spin model. Using the parameters $U = |t|$, $t_3 = 0.1t$ and $t = -2.7eV$, we depict the magnetization of the ground state solution in Fig. 5 for the two considered geometries. From this figure it is clear that the Néel order is identical for both ratios $J_{\mathrm{FM}}/J_{\mathrm{AFM}}$, with

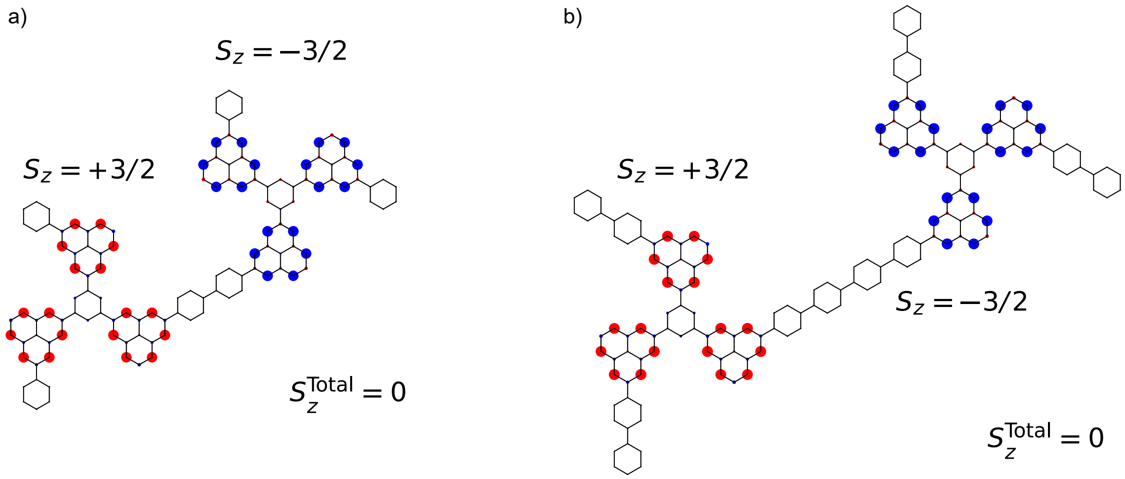

Figure 5: Magnetization maps of the ground state of the Hubbard Hamiltonian solved in mean-field, with $U = |t|$, $t_3 = 0.1t$ and $t = -2.7eV$. In panel a) we show the case with two benzene spacers, while in b) four benzene spacers are used. In both cases the total magnetization vanishes, and each trimer has $S_z = \pm 3/2$.

a vanishing magnetization in the unit cell, and oposite pollarization in the two trimers ($S_z = +3/2$ and $S_z = -3/2$).

Triplon spectrum

In this section we will give more details on the calculation of the triplon energy dispersion, based on the bond-operator formalism. First, we introduce the spin Hamiltonian for the

crystal described in the main text:

$$H = J_{\mathrm{AFM}} \sum_i \left[\boldsymbol{S}_3(\boldsymbol{r}_i) \cdot \boldsymbol{S}_4(\boldsymbol{r}_i) + \boldsymbol{S}_1(\boldsymbol{r}_i) \cdot \boldsymbol{S}_5(\boldsymbol{r}_i) + \boldsymbol{S}_2(\boldsymbol{r}_i) \cdot \boldsymbol{S}_6(\boldsymbol{r}_i) \right]$$
$$+ J_{\mathrm{FM}} \sum_i \left[\boldsymbol{S}_1(\boldsymbol{r}_i) \cdot \boldsymbol{S}_2(\boldsymbol{r}_i) + \boldsymbol{S}_1(\boldsymbol{r}_i) \cdot \boldsymbol{S}_3(\boldsymbol{r}_i) + \boldsymbol{S}_2(\boldsymbol{r}_i) \cdot \boldsymbol{S}_3(\boldsymbol{r}_i) \right]$$
$$+ J_{\mathrm{FM}} \sum_i \left[\boldsymbol{S}_5(\boldsymbol{r}_i) \cdot \boldsymbol{S}_6(\boldsymbol{r}_i + \boldsymbol{a}_1) + \boldsymbol{S}_5(\boldsymbol{r}_i + \boldsymbol{a}_2 - \boldsymbol{a}_1) \cdot \boldsymbol{S}_4(\boldsymbol{r}_i) + \boldsymbol{S}_6(\boldsymbol{r}_i + \boldsymbol{a}_2) \cdot \boldsymbol{S}_4(\boldsymbol{r}_i) \right] \tag{2}$$

where we assume $|J_{\mathrm{AFM}}| \gg |J_{\mathrm{FM}}|$. The sums over i run over all the unit cells of the crystal, whose position is given by $\boldsymbol{r}_i$; the vectors $\boldsymbol{a}_1$ and $\boldsymbol{a}_2$ are the primitive vectors of the crystal, and are represented in Fig. 6. In the same figure we label the spins in the unit cell from 1

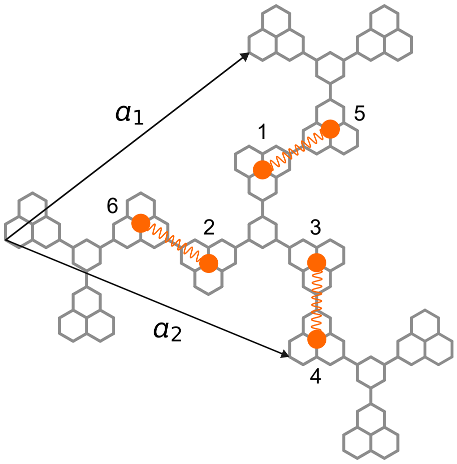

Figure 6: Schematic representation of the spin model used to compute the triplon dispersion. A spin-1/2 is assigned to each phenalenyl, which are strongly coupled via an antiferromagnetic exchange in pairs. Also depicted are the lattice vectors $\boldsymbol{a}_1$ and $\boldsymbol{a}_2$ used in the triplon calculation.

to 6.

Now, following Sachdev and Bhatt,[4–6] we introduce singlet and triplet bond operators

(assumed to be bososnic) for each pair of antiferromagnetically coupled spins

$$|s\rangle = s^\dagger|0\rangle = \frac{1}{\sqrt{2}}\left(|\uparrow\downarrow\rangle - |\downarrow\uparrow\rangle\right) \tag{3}$$

$$|t_x\rangle = t_x^\dagger|0\rangle = -\frac{1}{\sqrt{2}}\left(|\uparrow\uparrow\rangle - |\downarrow\downarrow\rangle\right) \tag{4}$$

$$|t_y\rangle = t_y^\dagger|0\rangle = \frac{i}{\sqrt{2}}\left(|\uparrow\uparrow\rangle + |\downarrow\downarrow\rangle\right) \tag{5}$$

$$|t_z\rangle = t_z^\dagger|0\rangle = \frac{1}{\sqrt{2}}\left(|\uparrow\uparrow\rangle + |\downarrow\downarrow\rangle\right) \tag{6}$$

where $|s\rangle$ stands for the formation of a singlet in the bond, and t_α with $\alpha = x, y, z$ refers to the formation of a triplet. The spins forming a given bond can be expressed in terms of these operators as

$$S_{i\alpha} = \frac{1}{2}\left(s^\dagger t_\alpha + t_\alpha^\dagger s - i\epsilon_{\alpha\beta\gamma} t_\beta^\dagger t_\gamma\right) \tag{7}$$

$$S_{j\alpha} = \frac{1}{2}\left(-s^\dagger t_\alpha - t_\alpha^\dagger s - i\epsilon_{\alpha\beta\gamma} t_\beta^\dagger t_\gamma\right) \tag{8}$$

with repeated indexes being summed over. In order to have physically meaningful results, a constraint of the form $s^\dagger s + t_\alpha^\dagger t_\alpha = 1$ must be enforced. In practice this is achieved through the introduction of a chemical potential μ, which fixes the number of bosons as 1 per bond. For two exchange coupled spins, i.e. $J\boldsymbol{S}_i \cdot \boldsymbol{S}_j$, the eigenstates are either a singlet with energy $-3J/4$ or a triplet with energy $J/4$. Hence, we write

$$J\boldsymbol{S}_i \cdot \boldsymbol{S}_j = -\frac{3J}{4} s^\dagger s + \frac{J}{4} t_\alpha^\dagger t_\alpha \tag{9}$$

Because in our unit cell we have three pairs of antiferromagnetically coupled spins, an additional index, ν, has to be attached to the bond operators to distinguish to which bond they are referring to. We shall use the following convention for the bond labels: a for spins $3-4$, b for $1-5$ and c for $2-6$, in agreement with the definition given in Fig. 6. To proceed with the calculation two approximation are introduced at this stage: i) we assume that the

number of singlets is much larger than the number of triplets, prompting us to replace the singlet operators by a number, s, representing the average singlet population at each bond, i.e. $\langle s\rangle \approx \langle s^\dagger\rangle \approx \bar{s}$, where in the limit $|J_{\mathrm{AFM}}| \gg |J_{\mathrm{FM}}|$ we expect $\bar{s}\sim 1$; ii) assuming a low triplet population, we neglect terms containing the product of more than two triplet operators, since these would contribute to triplet-triplet scattering which we assume to be negligible.

Using the aforementioned approximations and introducing the Fourier transform of the operators

$$t^\dagger_{\nu i\alpha} = \frac{1}{\sqrt{N}}\sum_{\boldsymbol{k}} t^\dagger_{\nu\boldsymbol{k}\alpha} e^{i\boldsymbol{k}\cdot\boldsymbol{r}_i} \tag{10}$$

with $\nu = a, b, c$ we write the spin Hamiltonian in momentum space as

$$\begin{aligned} H = {} & 3N\left(-\frac{3}{4}J_{\mathrm{AFM}}\bar{s}^2 - \mu\bar{s}^2 + \mu\right) + \sum_{\nu=\mathrm{a,b,c}}\left(\frac{J_{\mathrm{AFM}}}{4} - \mu\right)\sum_{\boldsymbol{k}} t^\dagger_{\nu\boldsymbol{k}\alpha} t_{\nu\boldsymbol{k}\alpha} \\ & + \frac{J_{\mathrm{FM}}\bar{s}^2}{4}\sum_{\boldsymbol{k}}\left[t_{b\boldsymbol{k}\alpha}t_{c-\boldsymbol{k}\alpha}\left(1+e^{i\boldsymbol{k}\cdot\boldsymbol{a}_1}\right) + t_{b\boldsymbol{k}\alpha}t^\dagger_{c\boldsymbol{k}\alpha}\left(1+e^{i\boldsymbol{k}\cdot\boldsymbol{a}_1}\right) + \mathrm{h.c.}\right] \\ & + \frac{J_{\mathrm{FM}}\bar{s}^2}{4}\sum_{\boldsymbol{k}}\left[t_{b\boldsymbol{k}\alpha}t_{a-\boldsymbol{k}\alpha}\left(1+e^{-i\boldsymbol{k}\cdot(\boldsymbol{a}_2-\boldsymbol{a}_1)}\right) + t_{b\boldsymbol{k}\alpha}t^\dagger_{a\boldsymbol{k}\alpha}\left(1+e^{-i\boldsymbol{k}\cdot(\boldsymbol{a}_2-\boldsymbol{a}_1)}\right) + \mathrm{h.c.}\right] \\ & + \frac{J_{\mathrm{FM}}\bar{s}^2}{4}\sum_{\boldsymbol{k}}\left[t_{c\boldsymbol{k}\alpha}t_{a-\boldsymbol{k}\alpha}\left(1+e^{-i\boldsymbol{k}\cdot\boldsymbol{a}_2}\right) + t_{c\boldsymbol{k}\alpha}t^\dagger_{a\boldsymbol{k}\alpha}\left(1+e^{-i\boldsymbol{k}\cdot\boldsymbol{a}_2}\right) + \mathrm{h.c.}\right]. \end{aligned} \tag{11}$$

This Hamiltonian can now be expressed in the compact form $H = \sum_{\boldsymbol{k}} \Psi^\dagger_{\boldsymbol{k}}\mathcal{H}_{\boldsymbol{k}}\Psi_{\boldsymbol{k}}$, with

$$\Psi^\dagger_{\boldsymbol{k}} = \left(t^\dagger_{a\boldsymbol{k}\alpha}, t^\dagger_{b\boldsymbol{k}\alpha}, t^\dagger_{c\boldsymbol{k}\alpha}, t_{a-\boldsymbol{k}\alpha}, t_{b-\boldsymbol{k}\alpha}, t_{c-\boldsymbol{k}\alpha}\right),$$

and can be diagonalized with a paraunitary transformation. The values of the parameters μ and $\bar{s}$ are determined by solving the saddle-point equations $\langle\frac{\partial H}{\partial\mu}\rangle = 0, \quad \langle\frac{\partial H}{\partial\bar{s}}\rangle = 0$, where the averages are taken over the triplon vacuum. These relations give a set of self-consistent equations that must be solved iteratively, and whose solution determines μ, $\bar{s}$ and the triplon energy dispersion.

Classical estimate of $\mathcal{J}^{\mathbf{eff.}}_{S=3/2}$

In order to obtain an analytical estimate the of the coupling between the effective $S = 3/2$ spins, in the limit where $|J_{\mathrm{FM}}|/J_{\mathrm{AFM}}$ is very large, we consider two classical spin models. First, a dimer composed of 2 spin $S = 3/2$, with coupling $\mathcal{J}^{\mathrm{eff.}}_{S=3/2}$ for which the energy difference between FM and AFM solutions is

$$E_{\mathrm{FM}} - E_{\mathrm{AFM}} = 2\mathcal{J}^{\mathrm{eff.}}_{S=3/2}S^2 = \frac{9}{2}\mathcal{J}^{\mathrm{eff.}}_{S=3/2} \tag{12}$$

We now consider a model of 6 spins $s = 1/2$, arranged as in the star lattice dimer and calculate the energy difference between the two colinear configurations that we can have where the spins in the same trimer are parallel to each other, namely, the FM and AFM configurations. We obtain

$$E_{\mathrm{FM}} - E_{\mathrm{AFM}} = 2J_{\mathrm{AFM}}s^2 = \frac{J_{\mathrm{AFM}}}{2}. \tag{13}$$

Combining these two equations we obtain

$$\mathcal{J}^{\mathrm{eff.}}_{S=3/2} = \frac{J_{AF}}{9} \tag{14}$$

Comparison between the $S = 3/2$ and $S = 1/2$ Star lattice model in the strong ferromagnetic limit

In Fig. 7 we compare the energies obtained by diagonalizing the spin-1/2 model in the star lattice, and the spin-3/2 Heisenboerg model in the honeycomb lattice for the cluster considered in the main text using different ratios of $J_{\mathrm{FM}}/J_{\mathrm{AFM}}$. Already for $J_{\mathrm{FM}} = -5J_{\mathrm{AFM}}$ we see a decent agreement in the spectrum, but as we raise the value of this ratio, we obtain an excellent overlap of both energies and therefore the system behaves effectively as

a $S = 3/2$ system.

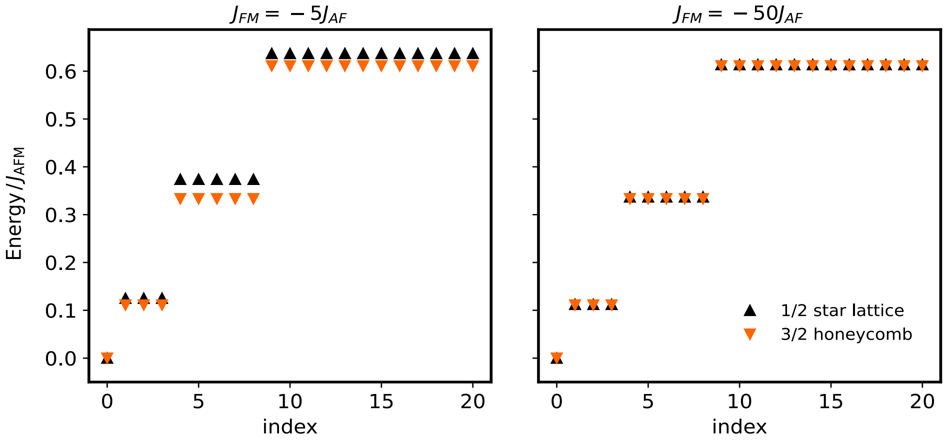

Figure 7: Low energy spectra of both the star lattice with $S = 1/2$ and the Heisenberg model with $S = 3/2$ using exact diagonalization. Comparison of different values of the ratio $J_{\mathrm{FM}}/J_{\mathrm{AFM}}$, with $J_{\mathrm{FM}} = -5J_{\mathrm{AFM}}$ on the left and $J_{\mathrm{FM}} = -50J_{\mathrm{AFM}}$ on the right. We can see here how as $J_{\mathrm{FM}}/J_{\mathrm{AFM}} \to \infty$, both spectra exhibit higher agreement.

References

(1) Catarina, G.; Henriques, J. C. G.; Molina-Sánchez, A.; Costa, A. T.; Fernández-Rossier, J. Broken-symmetry magnetic phases in two-dimensional triangulene crystals. *Physical Review Research* **2023**, *5*, 043226.

(2) Ortiz, R.; Boto, R. Á.; García-Martínez, N.; Sancho-García, J. C.; Melle-Franco, M.; Fernández-Rossier, J. Exchange rules for diradical π-conjugated hydrocarbons. *Nano Letters* **2019**, *19*, 5991–5997.

(3) Jacob, D.; Fernández-Rossier, J. Theory of intermolecular exchange in coupled spin-1 2 nanographenes. *Physical Review B* **2022**, *106*, 205405.

(4) Sachdev, S.; Bhatt, R. N. Bond-operator representation of quantum spins: Mean-field

theory of frustrated quantum Heisenberg antiferromagnets. *Physical Review B* **1990**, *41*, 9323.

(5) Gopalan, S.; Rice, T. M.; Sigrist, M. Spin ladders with spin gaps: A description of a class of cuprates. *Physical Review B* **1994**, *49*, 8901.

(6) Normand, B.; Rice, T. M. Electronic and magnetic structure of $LaCuO_{2.5}$. *Physical Review B* **1996**, *54*, 7180.
